# Adenine base editor for knockout of proteins: A practical guide from design to analysis with updated MultiEditRbatch

**DOI:** 10.1016/j.omtn.2026.102908

**Published:** 2026-03-18

**Authors:** Ella J. Eaton, Bryce J. Wick, Jeremy S. Chacón, Amy J. Wang, Mitchell Kluesner, Jackson T. Barnes, Bibekananda Kar, Minjing Wang, Matthew J. Johnson, Joseph G. Skeate, Beau R. Webber, Branden S. Moriarity

**Affiliations:** 1 Department of Pediatrics, University of Minnesota, Minneapolis, MN, USA; 2 Masonic Cancer Center, University of Minnesota, Minneapolis, MN, USA; 3 Center for Genome Engineering, University of Minnesota, Minneapolis, MN, USA; 4 Minnesota Supercomputing Institute, University of Minnesota, Minneapolis, MN, USA; 5 Molecular and Cellular Biology Graduate Program, University of Washington, Seattle, WA, USA; 6 Human Biology Division, Fred Hutchinson Cancer Research Center, Seattle, WA, USA; 7 Department of Immunology, Mayo Clinic, Rochester, MN, USA

**Keywords:** MT: RNA/DNA editing, knockout, CRISPR-Cas9, primary immune cell, splice signal disruption, sanger sequencing analysis, adenine base edit

## Abstract

Adenine base editors (ABEs) are a promising yet underutilized tool for inducing protein knockout compared to Cas9 nuclease, owing in part to a lack of user-friendly platforms for reagent design and implementation. Here, we present a comprehensive workflow to achieve high-efficiency gene knockouts with ABE as an alternative to Cas9 nuclease-based approaches. This includes optimized guide RNA (gRNA) design using SpliceR, a web-based application, followed by genomic and functional validation of ABE-mediated knockouts for several target genes. We validated gRNAs for 14 immunologically relevant targets, using both NGG and NG PAM compatible ABE8e-variants in primary immune cells. To facilitate data analysis, we developed MultiEditRbatch, an updated version of MultiEditR as a user-friendly analysis tool with addition of batch mode for high-throughput analysis of Sanger sequencing data. MultiEditRbatch is available as a web-based application and an R package, enabling robust assessment of base editing outcomes.

## Graphical abstract

**Figure:**
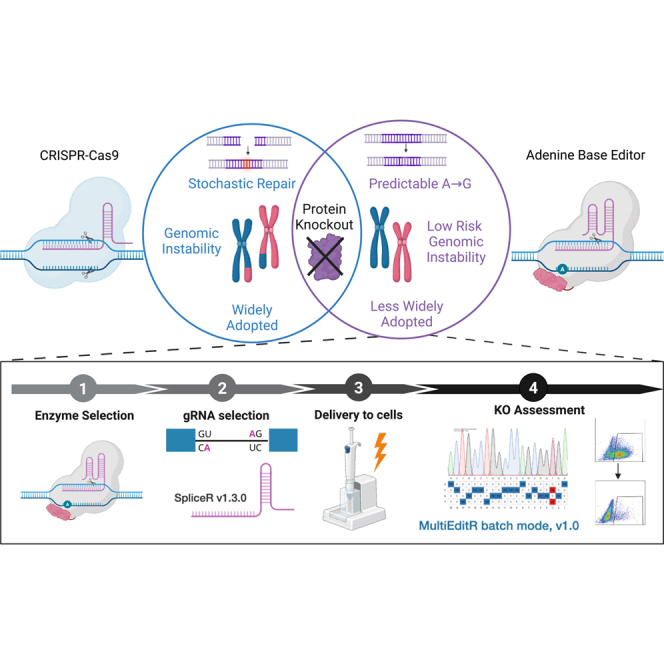

## Teaser

Moriarity and colleagues present a pipeline for implementation of adenine base editor (ABE) gene knockout tailored to researchers currently using CRISsPR-Cas9. ABE is better suited for multiplex knockout with significantly lower risk of chromosomal instability. MultiEditRbatch is introduced with batch mode; available as a web application and downloadable R package.

## Introduction

CRISPR-Cas9-mediated gene knockout is an essential tool for exploring gene function, interactions, and clinical applications such as cellular therapies. Cas9 works by using a guide RNA (gRNA) to create a targeted double-stranded break (DSB) in DNA, which is repaired through endogenous pathways such as homologous recombination or the error-prone non-homologous end joining (NHEJ).^1^ NHEJ can introduce insertions or deletions (indels) that disrupt gene expression, typically through frameshift mutations that alter amino acid sequences or trigger mRNA degradation via nonsense-mediated decay.^2^^,^^3^^,^^4^ The efficiency of Cas9 stems from its persistent cleavage of DNA until the gRNA target sequence is disrupted, reducing further binding and cleavage. To support this technology, numerous computational pipelines have been developed for gRNA design, off-target prediction, and analysis.^5^^,^^6^

Despite its broad utility, Cas9-mediated gene knockout has limitations. The reliance on NHEJ introduces stochastic editing outcomes, as indels vary depending on the DSB target site sequence context, potentially leading to suboptimal knockout efficiencies.^7^^,^^8^ While single or double nucleotide indels often cause frameshifts that effectively disrupt gene function, indels in multiples of three may preserve the open reading frame, leaving protein function intact. Additionally, the stochastic nature of NHEJ-driven repair can result in inconsistent outcomes, complicating experimental reproducibility. These challenges highlight the need for careful gRNA design and analysis to optimize knockout efficiency and minimize off-target effects.

Additionally, some cell types are not amenable to Cas9 knockouts due to increased sensitivity to DNA damage.^9^ For example, hematopoietic stem cells are highly sensitive to the double-strand breaks (DSBs) induced by Cas9.^10^ These DSBs activate TP53, which reduces viability of edited cells and significantly decreases long-term engraftment potential.^11^ Cell types with deficiencies in DNA damage repair genes are also less suited for Cas9 knockouts due to their increased risk for genomic instability, which is exacerbated by DSB induction.^8^ This instability of unprotected double stranded DNA, which is generally resolved by indel formation, can result in chromosomal translocations or loss. Often, Cas9-edited clones are not karyotyped, which can lead to inconsistent or inaccurate results downstream due to losses or translocations. The risk of chromosomal translocations is significantly increased if more than one gene is targeted with Cas9 simultaneously.^12^ To manipulate the genome without DSB induction, adenine and cytosine base editors (CBEs) were developed to utilize the programmable targeting of Cas9 while negating its nuclease activity.^13^

Adenine base editor (ABE), first described in 2017, generally does not induce DSBs (<1%) and is therefore a favorable alternative to Cas9 for single and multiplex gene knockouts.^12^^,^^14^ The initial ABE enzyme was comprised of a catalytically dead Streptococcus pyogenes Cas9 (spCas9) fused to a deaminase domain that facilitates the conversion of adenine (A) to inosine (I) within a defined editing window. Inosine is recognized as analogous to guanine (G) by the endogenous cell machinery, including RNA and DNA polymerases. Further engineering of ABEs, such as replacing the dead Cas9 with a Cas9 nickase (D10A), and phage-assisted continuous evolution (PACE) has led to ABE enzymes with greatly improved functionality. Current iterations of ABE use a nicking Cas9 domain to create a single-strand break in the DNA, which biases repair to match the deaminated strand.^15^ ABE8e, a variant published in 2020, exhibits a 590-fold increase in activity due to numerous mutations in the TadA deaminase domain.^16^ The area of genomic DNA that is accessible to the deaminase domain is called the editing window, which is on the opposite end of the gRNA sequence from the protospacer adjacent motif (PAM). Throughout this manuscript, we number the positions of the gRNA sequence 1–20 with 20 being closest to the PAM. The canonical editing window of ABE, positions 4–9 (11–16 nucleotides upstream from the PAM), has also been refined by altering the access of the deaminase domain to the target DNA.^17^ Further, evolution of Cas9 has yielded mutations that alter PAM preference and dependence; this reduced dependence on stringent PAMs increases the frequency of targetable sites using ABE.^18^^,^^19^^,^^20^ As a result of these alterations, ABE editing is now more efficient, precise, and modular with countless variants that can be selected for the unique requirements of each target.^15^^,^^21^^,^^22^^,^^23^ ABE has been widely used to model single nucleotide variants (SNVs) and revert disease-causing alleles to restore function^24^^,^^25^ and to install gain of function edits, such as the dominant-negative alleles of FAS cell surface death receptor to increase cytotoxicity of CAR-T cells^26^ and non-cleavable CD16a to improve antibody dependent cellular cytotoxicity (ADCC).^27^

Compared to Cas9 nuclease, ABE produces a more precise and predictable outcome (i.e., indel vs. A–G editing). However, ABE8e can cause indel formation by way of non-homologous or microhomology-mediated end joining, albeit at astonishingly lower rates than Cas9 (<1%).^11^^,^^28^ The single-strand break and inosine intermediates of ABE editing are resolved in one of two ways: restoration of original nucleotide by short-patch single-strand break repair followed by base excision repair, or more frequently, conversion of the A·T dinucleotide to G·C by long-patch single-strand break repair, non-canonical mismatch repair, or replication, where endogenous machinery treats inosine as guanine.^29^ Aside from rare indel formation, ABE editing results in two possible outcomes: the wild-type A·T or the edited G·C. Due to ABE’s low rate of DSB induction and subsequent indel formation, the p53 DSB response is largely not activated and in the case of hematopoietic stem cells, engraftment potential is less impacted by ABE.^11^ ABE has also been shown to induce translocations at a significantly lower rate than Cas9 when multiple genes are targeted.^30^ For multiplex knockout experiments, ABE poses a significantly lower threat to genomic integrity as well as a more precise editing outcome than Cas9.

**Figure 1:**
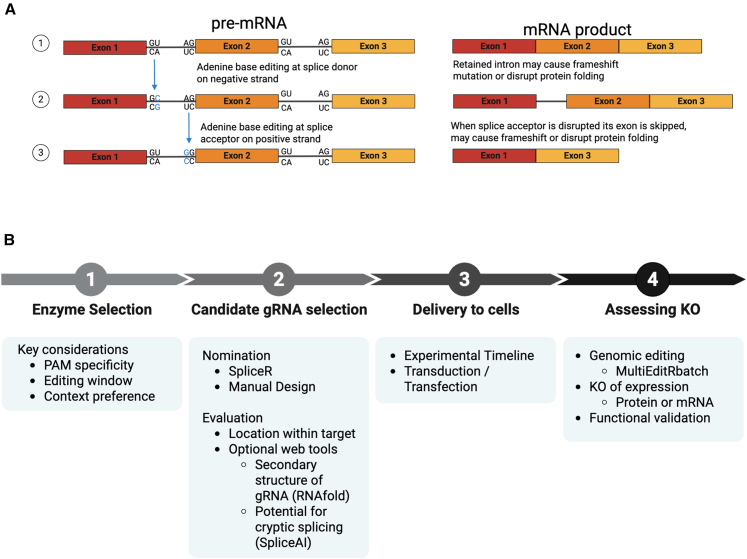
ABE-mediated knockout mechanism and pipeline (A) Mechanism of ABE knockout: editing of splice signals and the resulting mRNA products. (1) Normal splicing brings exons 1, 2, and 3 together while excluding the introns. (2) Editing the GU splice donor signal by targeting adenine on the negative strand results in a retained intron. (3) Editing the AG splice acceptor signal by targeting adenine on the positive strand result in a skipped exon. Disruptions to splice sites may cause frameshift mutations or disrupt protein folding. (B) Proposed pipeline for implementing ABE knockouts.

A number of approaches can be used to knock out gene function using ABE.^31^ Mutation of the ATG start codon can disrupt initiation of translation and either prevent translation or cause initiation to occur at a later methionine in the RNA transcript, which can result in truncated or missense peptides.^32^ Additionally, splicing signals can be edited to disrupt mRNA processing, resulting in skipped exons, retained introns, or frameshift mutations (Figure 1A). Disruption of splice sites can lead to nonsense-mediated decay of the mRNA, translation of a nonfunctional protein, truncated protein, or a peptide with a vastly different amino acid sequence than the unedited gene that is unlikely to perform the same function as the original. Alternatively, CBEs can be used to introduce premature stop codons, which can cause early termination of translation and result in nonsense-mediated decay of the mRNA.^4^^,^^12^

Despite the advantages of ABE for precise gene knockout, particularly multiplex knockouts, groups outside of the genome engineering field tend to use Cas9 nuclease. One possible explanation for this is the lack of available user-friendly tools to aid in design, experimentation, and analysis for base editor knockout like those available for Cas9. Computational tools for base editors are limited compared to the available curated Cas9 workflows offered by companies such as Synthego, Integrated DNA Technologies, Takara, and others. Here, we have compiled resources for ABE-mediated gene knockout including gRNA design tools, sources of reagents, experimental workflows, and analysis tools to increase accessibility of ABE- mediated gene knockout for the larger research community. Thus, this work aims to provide an easy-to-follow pipeline for deploying ABE for gene knockout (Figure 1B). Below, we provide resources for experimental design and downstream analysis of ABE-mediated knockout. Representative results for 15 target genes of immunological interest, with 14 successfully validated gRNA sequences, demonstrate the applicability of this pipeline for other fields. Additionally, we present an updated analysis tool for all applications of base editing: MultiEditRbatch. MultiEditRbatch is particularly suited for this pipeline created for the wider research community as it analyzes Sanger sequencing data for editing analysis, which is more accessible and cost-effective than next-generation sequencing for many research groups. This program, built upon EditR and MultiEditR, offers an improved user interface, more descriptive error handling, and the addition of batch mode for analysis of multiple samples at once.^33^^,^^34^ Taken together, this work will provide a practical guide for researchers new to using base editors and an exciting update of a widely used analysis tool for those experienced in base editing.

### ABE-mediated gene knockout protocol

#### Base editor enzyme selection

Determine which ABE variant will be used in the experiment before designing gRNAs. The PAM variant (NG or NGG in the examples below), and deaminase variant (TadA8e here) are relevant to gRNA design to determine PAM accessibility and editing window, respectively. In general, less stringent PAM variants can produce more off-target editing, which may be undesirable depending on the experimental context.^35^ If designing gRNA for eventual multiplex editing, only one base editor variant should be used at once within a single experimental condition. For example, to knock out two targets in one population of cells, one with an NG PAM and one with NGG, ABE8eNG should be used. NGG gRNAs are largely compatible with NG enzymes, but the inverse is not true.

Like Cas9, ABE is directed to a target site by a gRNA with 20 nucleotides of homology upstream of the PAM in genomic DNA. ABE8e has an “editing window,” the nucleotides accessible for deamination, of positions 4–9 in the protospacer (11–16 bases upstream from the PAM).^16^ The editing window depends on the ABE variant utilized. BE-keeper by Huang et al. is a useful tool for selecting the right variant for a given project, web page (https://be-keeper.broadinstitute.org/).^36^ For general ABE gRNA design the same group’s BE-hive web tool (https://www.crisprbehive.design/) is useful for predicting editing efficiency based on the sequence context preferences of each base editor.^37^

#### Candidate gRNA selection

**Figure 2:**
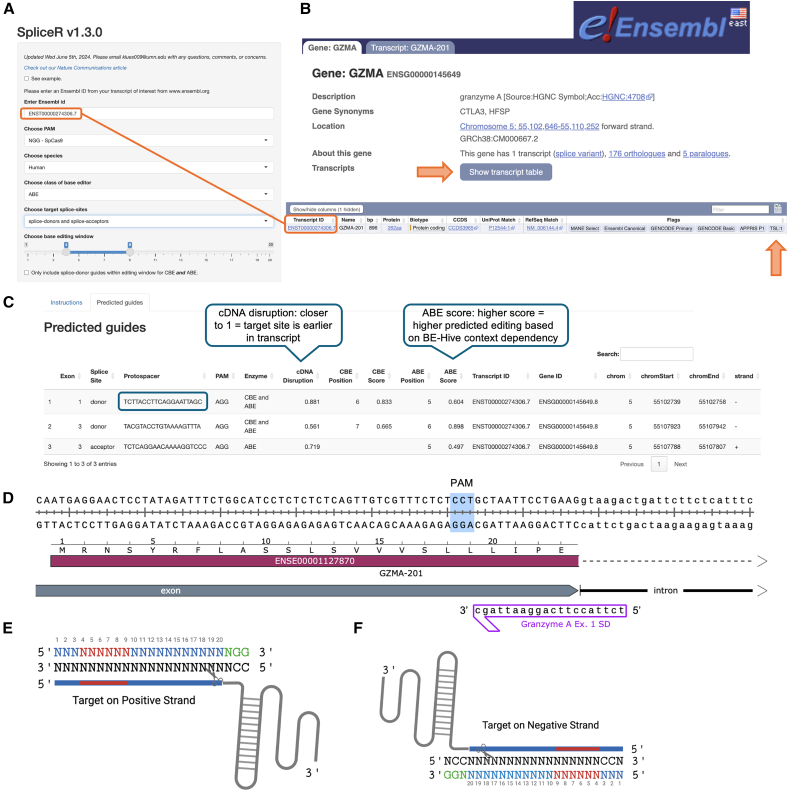
Guide RNA design (A) SpliceR v.1.3.0 interface with *GZMA* transcript ID. (B) Ensembl *GZMA* human gene page. Shown transcript table button is denoted with an orange arrow. Below are the transcript table results with the TSL1 designation denoted by an orange arrow. The transcript ID used for SpliceR input is denoted with an orange box. (C) SpliceR v.1.3.0 output is found within the “predicted guides” tab. Candidate gRNA is denoted with a teal box. (D) Genomic sequence containing exon 1 of *GZMA*. The indicated gRNA from (C) is denoted in purple and binds to the negative strand. The AGG PAM is highlighted in blue. Position 5 of the gRNA disrupts the GT splice donor by converting it to GC. (E and F) Visual reference for strand orientation. Green nucleotides represent the PAM, blue nucleotides represent protospacer, and red nucleotides represent the editing window of ABE8e within the protospacer. Scissors represent the strand nicked by Cas9 nickase.

The key to successful base editor-mediated gene knockout is gRNA design and screening.^31^ We suggest screening 3–6 gRNAs per gene target to maximize chances of success. This suggestion is based on the current capabilities of prediction software and our lab’s experience. Previous publications have described ABE-mediated mutation of AUG start codons for gene knockout,^32^ but design of gRNAs was performed manually. SpliceR (https://moriaritylab.shinyapps.io/splicer/) is a program developed by our group that identifies and scores potential gRNA sequences for gene knockout by splice site disruption.^31^ This work found that splice donors were generally more effective than splice acceptors. The user interface of SpliceR is shown in Figure 2A.

##### SpliceR gRNA design

SpliceR generates candidate gRNAs based on a user-provided Ensembl transcript IDs (https://useast.ensembl.org/index.html) (Figure 2B). Each candidate gRNA is assigned two numerical scores: a cDNA disruption score and an ABE/CBE score (Figure 2C). The cDNA disruption score predicts the likelihood of this edit resulting in non-functional mRNA based on the proximity of the edit to the beginning of the transcript, as our studies have found that targeting splice sites closer to the beginning of the transcript will result in more disruption.^31^ The ABE/CBE score uses data from BE-Hive, including the context specificity of enzymes listed in the base editor class dropdown and relative editing efficiency at positions within the editing window.^37^

Design of gRNA protospacer candidates with SpliceR:

1. Navigate to Ensembl.org to find the transcript ID of the isoform of the target gene. In the search tab, enter the desired species and the name or abbreviation of your gene of interest. Select the gene of interest with the notation (human gene) from the results list and click “show transcript table.” This table lists details of each known isoform of the gene. In the column labeled “flags” identify the TSL1 designation (transcript support level 1) to select transcripts that are validated by the type and quality of alignments used to annotate the transcript. If there are multiple isoforms labeled TSL1, choose the predominant transcript or use all TSL1 transcripts for gRNA design and select gRNAs that bind to all TSL1 transcripts (Figure 2B).
2. Navigate to SpliceR and input the transcript ID (Figure 2A). Choose the desired PAM variant and class of base editor you will be using downstream in the dropdowns. Choose desired target splice-sites from the dropdown; note, splice donors tend to be more effective, but splice acceptors can work just as efficiently depending on the individual transcript.^27^ Finally, select the base editing window using the slider bar. The default is positions 4–9 but you can narrow results using this tool to specify the ideal window for your specific editor enzyme.
3. Select the “predicted guides” tab to view the list of generated gRNAs (Figure 2C). We recommend ordering 3–6 guides to test initially and have found that one typically produces sufficient editing (i.e., >75% editing). Choose the gRNAs based on the exon target (earlier is better), cDNA disruption score (higher is better), and CBE/ABE score (higher is better). If few or no gRNAs are generated, consider using an editor with a less stringent PAM.

##### Manual gRNA design

Although less ideal, base editor gRNAs can be manually designed based on the genomic DNA sequence annotated with exons. It is helpful to view both strands of the genomic DNA at the target site to consider potential gRNAs on the opposite DNA strand. After the target base is selected, a PAM must be identified. Figures 2D–2F provides visual aids for orienting around the PAM and designing a gRNA. For spCas9-based editors such as ABE8e, the PAM is always 3′ to the target base on the same DNA strand. Protospacer numbering starts on the 3′ end of the 20-base gRNA. Base editors use Cas9 D10A nickase, which creates a single-strand break on the non-target DNA strand.

##### Target-specific considerations

**Figure 3:**
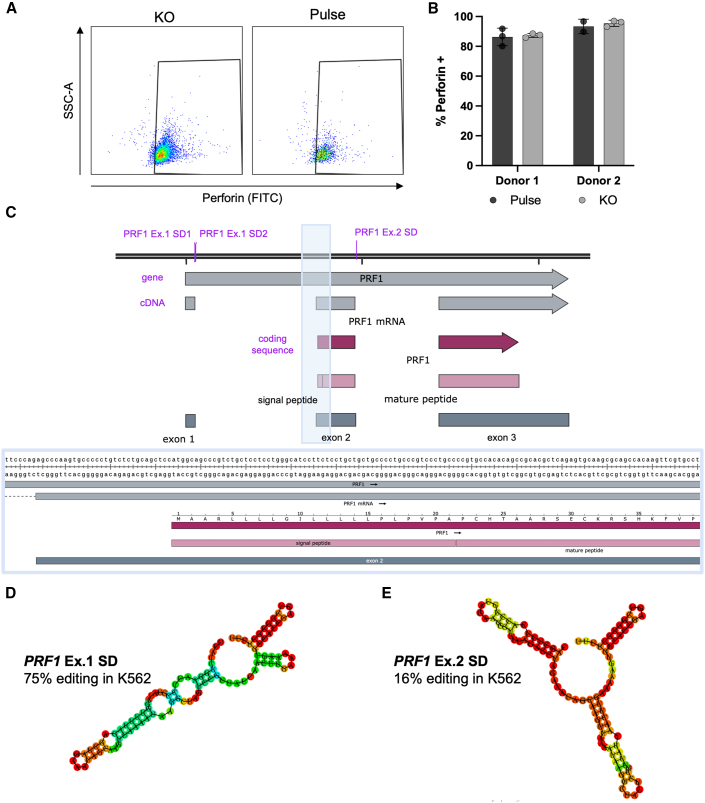
Failed guide validation of perforin (A) Representative flow plots of Perforin expression. Gating scheme: lymphocytes, single cells, live, CD3+. (B) Perforin expression from 2 independent donors of primary T cells edited with *PRF1* Ex.1 SD gRNA (KO) or unedited controls (pulse). Points represent n=3 technical replicates of intracellular staining and error bars denote standard deviation. (C) Genomic region of human *PRF1*. Three gRNAs were designed to target human *PRF1*, noted in purple. Perforin undergoes post-translational cleavage resulting in a shorter mature peptide that does not include exon 1. (D) The two gRNAs targeting the exon 1 splice donor resulted in no functional knockout by ICS-FC. (E) The other gRNA, targeting exon 2 splice donor resulted in low editing efficiency, potentially due to issues with secondary structure preventing association with the ABE8e enzyme.

Beyond the SpliceR cDNA disruption score output, it can also be helpful to consider functional domains of the target protein, which the SpliceR algorithm does not account for currently. It is prudent to check for other isoforms of the protein, which may use different translational start sites or alternative splicing; it may also be helpful to annotate the genomic DNA of your target with the nominated gRNAs and compare them to the cDNA or mRNA transcripts, as seen in Figure 3C. UniProtKB (https://www.uniprot.org/uniprotkb) can be helpful for determining important domains of target protein for rational design of gRNAs. For example, gRNAs targeting the exon 1 splice donor of *PRF1* did not result in a functional knockout, despite high editing efficiency (Figure 3A,B). This is likely due to the absence of exon 1 in the mature peptide after cleavage of the N terminus.^38^ Exon 2 of *PRF1* contains its own start codon, so disruption of exon 1 does not result in knockout of expression or function (Figure 3C). While the earlier domains are more likely to result in nonsense mediated decay of mRNA, targeting later domains with important folding or trafficking properties can also result in a functional knockout of the gene of interest.^39^ Due to the multiple factors that can contribute to an unsuccessful knockout gRNA and lack of comprehensive predictive tools, we suggest screening at least 3 gRNAs per target and leveraging tools such as UniProtKB, RNAfold, and SpliceAI to inform selection of gRNAs.

##### Web tools for informing gRNA design RNAfold and SpliceAI (optional)

Currently available software is proficient in gRNA nomination, but their predictive power is still lacking because they do not incorporate other metrics for gRNA success, such as secondary structure.^40^ A web tool called RNAfold (http://rna.tbi.univie.ac.at/cgi-bin/RNAWebSuite/RNAfold.cgi) is helpful to analyze gRNAs for undesirable secondary structure.^41^^,^^42^^,^^43^ When using this tool, it is critical to add the constant scaffold region of the gRNA directly after the 20 nucleotide protospacer sequence. Ensure that all 3 stem loops of the scaffold region are present, with the same sequences protruding from the loop. An example of gRNA secondary structure is shown in Figure 3, where the exon 1 gRNA results in successful editing of the genomic DNA, while the exon 2 guide edits at a low rate, potentially due to the inaccessibility of the protospacer to bind to genomic DNA and the disruption of normal stem loops that facilitate binding to the Cas9 enzyme (Figures 3C and 3D).

SpliceAI (https://spliceailookup.broadinstitute.org/) is a web tool particularly helpful for determining the likelihood of a successful knockout due to splice signal editing. Input the genomic location of the edit made by the gRNA to validate that a loss of splicing will occur.^44^ Additionally, this tool can identify possible cryptic splice sites that may be activated upon editing that would cause alternative splicing resulting in a potentially functional target.

#### Delivery of ABE and gRNA to cells

##### Transfection or transduction

ABE enzymes and gRNAs can be delivered in multiple forms based on the goals of the experiment and cell type.^36^ Many immortalized cell lines tolerate DNA plasmid expression of ABE and gRNA(s) well and numerous ABE variants and single guide RNA (sgRNA) expression vectors are available on Addgene (https://www.addgene.org/). Unfortunately, there are very few off-the-shelf ABE mRNA or proteins available commercially at this time. This is possibly due to the rapid evolution of the enzyme and demand for specialized variants, instead of a single one-size-fits-all version like Cas9 nuclease. The additional step of producing purified protein may be cost-prohibitive due to the insolubility of the protein, so any purchased ABE enzyme in mRNA or protein format is usually custom-ordered.^45^ One way to circumvent this issue is to deliver both enzyme and gRNA(s) using non-integrating viral vectors, such as recombinant adeno-associated virus (rAAV), adenovirus, or virus-like particles.^46^^,^^47^^,^^48^^,^^49^ Alternatively, ABE mRNA delivery is widely used for transient base editor expression. For many cell types, the mMessenger mMachine T7 kit (Thermo Fisher Scientific) produces mRNA that works successfully.^50^^,^^51^ However, for highly sensitive cells, such as primary immune cells, chemically modified mRNA with CleanCap AG is best for maximum expression and minimal intracellular nucleic acid recognition.^52^ Conveniently, commercial gRNA products sold for spCas9 editing are compatible with spCas9 base editors.^36^ Note, most commercially sourced Cas9 gRNAs use chemically modified nucleotides on each end to enhance stability and slow degradation.^53^ Some cell types, such as primary human natural killer (NK) cells and monocytes, may benefit from addition of RNase inhibitor (Millipore Sigma, Cat #3335399001) to reduce degradation of the mRNA and gRNA by endogenous RNases.^27^^,^^54^

##### Experimental timeline and considerations

**Figure 4:**
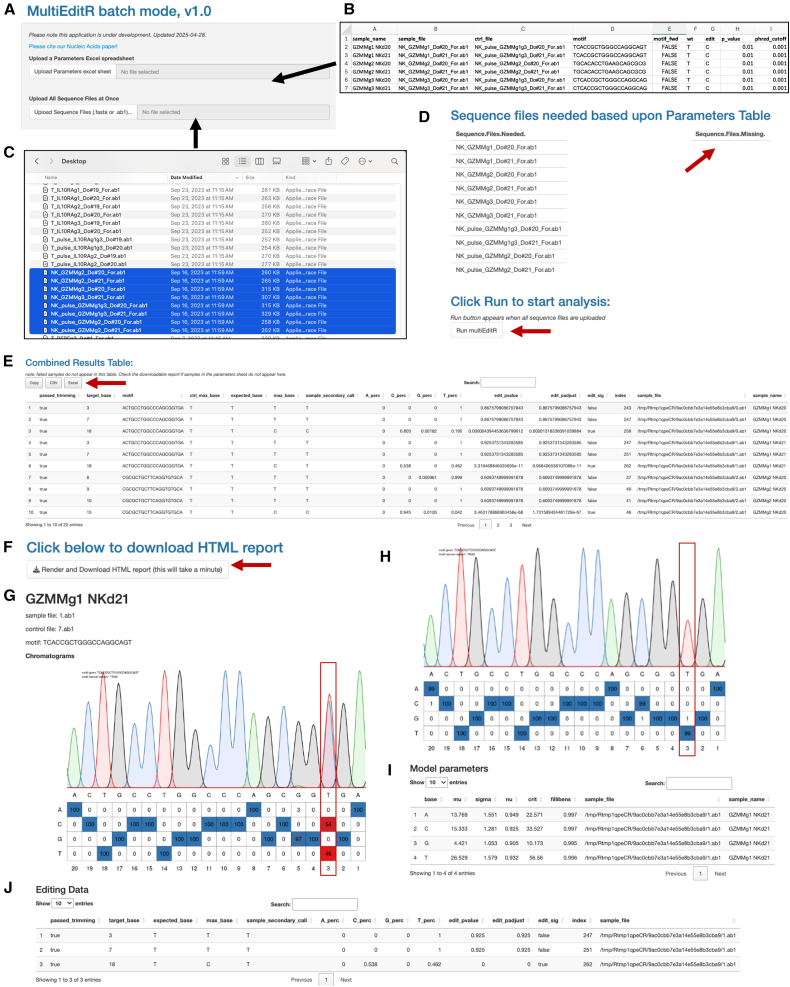
MultiEditR batch web interface (MultiEditRbatch) (A) Input for parameters spreadsheet (pictured in B) and sequence files (pictured in C). Parameters spreadsheet skeleton can be downloaded from the instructions page. All sequencing files (.ab1 or.fa format) must be selected and uploaded at once. (D) Confirmation that needed sequence files are present within the analysis tab. Arrows indicate empty “sequence files missing” list and “run MultiEditR” button. (E) Combined results table output after analysis is complete. This table shows all possible edits within the designated motifs. Arrow indicates buttons to copy or download data within this table. (F) Arrow indicates button to download HTML report, which includes data shown in (G–J). (G) Sample chromatogram within the given motif is always present for successfully analyzed samples. (H) Control sequence chromatogram is present only for .ab1 files when the motif is the same orientation in both sample and control. (I) Model parameters used for statistical tests in (E and J). (J) Editing data for all the indicated wt bases within the motif. In this example, the wt base is T and the edited base is C. Potential editing is detected at three positions, but only the edit at position 3 is deemed statistically significant.

Experimentally, ABE-mediated gene knockout requires a similar workflow to Cas9 knockouts. First, cells are transfected with ABE enzyme and gRNA, either as mRNA and gRNA or encoded in plasmids, via lipofection, cationic lipid-based transfection, electroporation, or transduction with a viral vector.^36^ After transfection or transduction, cells are cultured according to their normal protocol and samples are collected after 3–5 days.^36^ Always include a negative control, listed in Figures 3 and 4 as “pulse,” in which cells undergo the same treatment as edited conditions (electroporation, lipofection, etc.) but without gRNA. In sequencing experiments, pulse controls confirm that the protospacer sequence is present in the cells being edited and highlight any sequence variations from the reference genome. In experiments to validate knockout by expression of the target, the pulse control serves as a standard of expression in that cell type and can be used for normalization of data. In functional validation experiments, pulse controls serve as a positive control for target function.

#### Validating ABE-mediated gene knockout

Success of gene knockout can be assessed at the genomic, transcript, protein, and functional levels. Genomic DNA is isolated from the cells and used as a template for PCR amplification of the target sequence. Purified PCR amplicons undergo Sanger sequencing to determine editing outcomes. Additionally, cells can be evaluated for knockout of the target gene by assessing abundance of mRNA transcript by qPCR or protein knockout by western blot, flow cytometry, or target specific functional assays.

##### Genomic editing

Sanger sequencing is sufficient for most ABE knockout genomic analyses, especially when corroborated with expression or functional knockout data. Next-generation sequencing can be used for genomic analysis but is more costly and not necessary for most gRNA screening experiments. Numerous tools have been developed for analysis of Sanger sequencing traces for base-edited samples including BEAT (https://hanlab.cc/beat/),^55^ DECODR (https://decodr.org/),^56^ and EditR (https://moriaritylab.shinyapps.io/editr_v10/).^36^ An improved version of EditR, called MultiEditR v.1.1, was published in 2021, with changes to noise handling, which allow more accurate assessment of numerous edits within a single protospacer.^33^^,^^34^ MultiEditR can analyze RNA editing in addition to the analysis of DNA editing established by EditR. The incorporation of control files for each target allows for a more precise assessment of editing above 5%.

Here, we present an updated analysis tool using the same algorithm as MultiEditR. This new iteration, MultiEditRbatch, incorporates a batch mode for streamlined analysis of multiple sequence files and more descriptive messages to troubleshoot errors. MultiEditRbatch was developed with a special focus on ease-of-use for those who are familiar with CRISPR-Cas9 commercial workflows. The input parameters.xls file was based on the Synthego ICE (Inference of CRISPR Edits) analysis bulk input template. New parameters such as motif_fwd and phred_cutoff are defined in the instructions tab, which is the first page shown when navigating to the application website and in the readme file of the MultiEditRbatch R package. Results are aggregated into a downloadable HyperText Markup Language (.html) file, where individual samples can be analyzed in depth through the generated EditR plot, editing data table, and model parameters data. MultiEditRbatch (https://moriaritylab.shinyapps.io/multiEditR-Batch-Mode/), has been published as a web-based application for wider use. The complete R package can be downloaded from GitHub (https://github.com/jeremymchacon/multieditR_Package) for local analysis without a web connection.

##### Using MultiEditRbatch

A step-by-step guide for the MultiEditRbatch web application is shown in Figure 4, with the user interface (Figure 4A). To input data into the parameters file, refer to the genomic sequence of your target with gRNA and sequencing/PCR primers labeled. In each row, input a unique sample name and the sample and control file names (Figure 4B). The same control file can be used for multiple sample files. Next, enter the gRNA sequence (5’→3′) into the motif column. The motif_fwd column indicates the orientation of the gRNA on the sample sequence. To determine the orientation referencing the genomic sequence, determine whether the gRNA matches the top strand (+) or bottom strand (−). In the SpliceR application, the orientation is denoted in the strand column. Next, determine the direction of the sequencing primer. If the gRNA and the sequencing primer match the same strand, then motif_fwd is true. If the gRNA and the sequencing primer do not match the same strand, then motif_fwd is false. The algorithm matches the control file orientation automatically to the sample file. The wt and edit columns ask the user to specify the target base and what base it will be converted to upon editing. For use with ABE, when motif_fwd is true, wt will be A and edit will be G. When motif_fwd is false, wt will be T and edit will be C.

The phred score, sometimes referred to as a *Q* score, relates to the quality of sequencing data and represents the probability that the base called is incorrect. The phred cutoff default is 0.001, if Sanger sequencing files are of lower quality, the phred cutoff can be increased. Finally, the *p* value column asks the user to specify the *p* value threshold for determining an edit significant, the default is 0.01. Orientation of the EditR plot will vary depending on which strand is being edited, and which primer was used to sequence the amplicon. Figures 2E and 2F may help orient users to the protospacer numbering for analysis.

**Figure 5:**
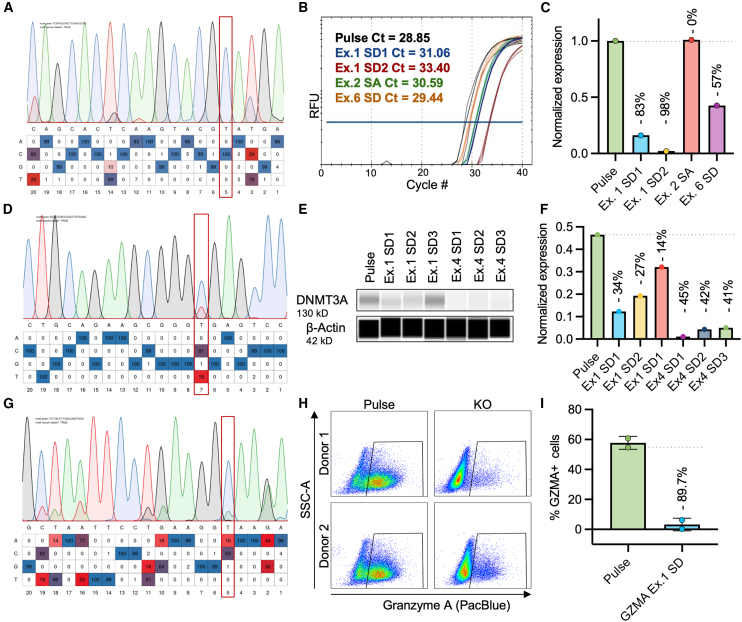
Validation of ABE-mediated gene knockouts (A–C) *NR4A3* gRNA validation. (A) EditR plot showing 100% conversion of T→C at position 9 with exon 1 splice donor 2 gRNA. (B) RT-qPCR amplification plot quantifying *NR4A3* transcript abundance after treatment with candidate gRNAs. (C) Bar graph depicting reduction in transcript abundance normalized to pulse control in 1 donor. (D–F) *DNMT3A* gRNA validation in 1 donor. (D) EditR plot showing 81% T→C at position 9 with exon 4 splice donor 3 gRNA. (E) Digital western blot for DNMT3A normalized using β-actin expression. (F) Bar graph depicting reduction in DNMT3A protein abundance compared to pulse control. (G–I) GZMA gRNA validation. (G) EditR plot showing 100% A→G editing at position 5 with exon 1 splice donor. (H) Flow cytometric validation of GZMA protein knockout in primary T cells. Stimulation conditions were incubated overnight with anti-CD3 and anti-CD28 monoclonal antibodies before intracellular staining. Plots showing GZMA expression of live, CD3+ cells. (I) Bar graph depicting proportion of GZMA+ cells. Points represent individual donors with error bars showing standard deviation. Dotted lines in (C, F, and I) shows average expression for unedited control in the same donor. Difference from the control, or percent loss of expression is noted above each bar and is equal to 1− (average of each group/average of control) ∗ 100.

Once the parameters file is complete, drop the .xls file into the interface (Figure 4A). Select all .ab1 and .fasta files at once and drop them into the lower box on the interface (Figure 4C). Under the analysis tab, the application will list the files needed and the files missing (Figure 4D). If anything appears in the sequence files missing list, you must select all of the same files plus any that were missing and drop them into the upload box again. When all files are uploaded, the Run MultiEditR button will appear. Once analysis is run, the combined results table will appear (Figure 4E). To see the chromatograms and individual sample analysis, render and download the HTML report (Figure 4F). Within this HTML, each sample will display the sample file chromatogram (Figure 4G), the control file chromatogram, when possible (Figure 4H), the model parameters used (Figure 4I), and the editing data (Figure 4J). Additional examples of sample chromatograms are shown in Figures 5A, 5D, and 5G.

Within the editing data table, all instances of the input for “wt” base within the provided motif will be displayed. Locate the position of the desired edit within the protospacer numbering in Figure 4G, to identify the relevant row of the editing data table. Within this row, the percentage of each base is displayed, along with the edit_pvalue (Figure 4I). For each candidate editing site within the motif window, MultiEditR reports the observed base frequencies and a raw *p* value quantifying whether the substitution exceeds background noise. No multiple-testing correction is applied in the current implementation. This matches the conditions used to calibrate MultiEditR in its original validation, where raw *p* values produced optimal sensitivity and specificity.^34^ Users should generally apply a significance threshold of *p* < 0.01 for higher sensitivity, or *p* < 0.001 (or 0.0001) when a very stringent, low-false-positive rate is desired.

##### Protein/mRNA expression knockout

Like other Cas9-based methods, the appropriate method for determining if a knockout eliminates expression will depend on the target. For example, targets in lower abundance such as nuclear receptor 4 A3 (NR4A3), a transcription factor, the sensitivity of quantitative reverse-transcription PCR (RT-qPCR) is ideal (Figure 5B). However, western blot may be ideal for intracellular targets present in abundance under normal physiological conditions—such as DNA methyltransferase 3 alpha (DNMT3A) (Figure 5E). Targets that are differentially expressed may need to be induced, such as stimulating primary T cells to induce granyzme A (GZMA) expression (Figure 5H). Expression can be normalized to an unedited control to determine the percent loss of expression as shown in Figures 5C, 5F, and 5I.

##### Functional validation

Validation of functional knockout is necessary when implementing any knockout strategy. Specifically, the use of splice site disruption for knockout must be verified due to the potential for alternative splicing that may retain function of the target while eliminating antibody epitopes or sequences of mRNA used for expression analysis. The most important screening tool for knockout is a target-specific assay of function. If screening guides in a cell line that will be later used in a different cell type, plan to perform functional validation experiments in a relevant model for that target.

## Results

Using the above-described pipeline, we designed and tested multiple candidate gRNAs for 15 independent targets of immunological interest (Table S1). Many guides were screened in K562 cells prior to primary immune cells to eliminate gRNAs with suboptimal editing efficiencies. Table 1 displays the highest performing gRNA for 14 of the 15 targets. Figure 5 displays representative results for knockouts of 3 gene targets: *NR4A3*, *DNMT3A*, and *GZMA* each are using a different method of expression knockout validation.

**Table 1:** Validated ABE KO gRNAs of immunological interest

Target	Description	Guide sequence primary cell tested	Exon target base	Genomic efficiency protein/mRNA loss
**NR4A1:** nuclear receptor subfamily 4 group A member 1	limits T cell function in response to NFAT signaling during chronic stimulation, partially through a reduction of NFkb and bZIP activity	GCTCTCACCTGTACGCCAGG	ex. 5 SD 1	100%, 100% (RT-qPCR)
		T cell	7	78% (RT-qPCR)
**NR4A2:** nuclear receptor subfamily 4 group A member 2	limits T cell function in response to NFAT signaling during chronic stimulation, partially through a reduction of NFkb and bZIP activity	TAATTGCAGGTCCAACCCAG	ex. 7 SA 1	100%, 100%
		T cell	8	84% (RT-qPCR)
**NR4A3:** nuclear receptor subfamily 4 group A member 3	limits T cell function in response to NFAT signaling during chronic stimulation, partially through a reduction of NFkb and bZIP activity	TCATACGTACTTGAGTGCTG	ex. 1 SD 2	100%, 100%
		T cell	9	98% (RT-qPCR)
**HPK1 (MAP4K1):** mitogen-activated protein kinase 1/hematopoietic progenitor kinase 1	negative regulator of TCR signaling, limiting strength and duration of TCR signaling through disrupting the SLP-76/LAT signalosomes	ATCACCAGAGATAACTCCCA	ex. 3 SD	100%, 99%
		T cell	4	98% (WB)
**SOCS1:** suppressor of cytokine signaling 1	negative regulator of cytokine signaling	CTCACCTGGCGGCGGGGCGC	ex. 1 SD 2	99%, 100%
		T cell	4	100% WB)
**RASA2:** RAS p21 protein activator 2	RasGAP that suppresses RAS signaling by increasing the rate of the hydrolysis of active RAS-GTP to RAS-GDP	ATTTACCTGAACCTCTGAAT	ex. 4 SD	100%, 100%
		T cell	5	99% (WB)
**CBL-B:** Cbl proto-oncogene B (Casitas B lymphoma-b)	E3 ubiquitin protein ligase which regulates CD28 and CTLA-4 signaling	ATTATACCTGCCATGCCGTA	ex. 9 SD	100%
		T cell	6	74% (WB)
**PTPN22:** protein tyrosine phosphatase non-receptor type 22	intracellular phosphatase which associates with CBL proteins and regulates TCR signaling	CTTACACAGGATACAGAGAA	ex. 6 SD	98%, 99%
		T cell	4	66% (WB)
**DNMT3A:** DNA methyltransferase 3 alpha	*de novo* DNA methyltransferase which catalyzes 5-methylcytosine methylation, regulates T cell effector functions and differentiation early after activation	GGACTCACCCGCTTCTGCAG	ex. 4 SD1	99%
		T cell	7	85% (WB)
**GZMB:** granzyme B	protease activated by cathepsin H that induces apoptosis by cleaving target cell procaspases	CACTCACCTGCATCTGCCCT	ex. 1 SD	81.4%, 94.2%
		T cell	6	88.5% (ICS-FC)
**GZMA:** granzyme A	protease that triggers a type of cell death called athetosis by interfering with the actin cytoskeleton	TCTTACCTTCAGGAATTAGC	ex.1 SD	82.6%, 79.6%
		T cell	5	89.7% (ICS-FC)
**GZMM:** granzyme M	protease which activates apoptosis by cleavage of numerous death substrates	TCACCGCTGGGCCAGGCAGT	ex. 2 SD	80.3%, 53.8%
		NK cell	3	51.7%, 17.8% (ICS-FC)
**GNLY:** granulysin	antibacterial toxin that ruptures membranes of intracellular bacteria	AGGCCTTACCTGGGTTGCCC	ex. 1 SD	98.8%, 99.5%
		T cell	8	76.6%, 92.4% (ICS-FC)
**IL-10Rα:** interleukin 10 receptor alpha	cell surface receptor for interleukin 10, mediates immunosuppressive signal to reduce inflammatory response	CCCCATACCGTGAAGTTTCC	ex. 4 SD	92%, 100%
		T cell	7	38.3%, 54.1% (ICS-FC)

Highest performing gRNA for each target. Cells with two values represent results from two individual donors. Protein/mRNA loss method of validation denoted in parentheses: western blot (WB), quantitative reverse transcriptase PCR (RT-qPCR), or intracellular staining and flow cytometry (ICS-FC).

NR4A3 is a nuclear receptor protein that limits T cell function in response to NFAT signaling during chronic stimulation.^57^ Four candidate gRNAs targeting the exon 1 and 6 splice donors or exon 2 splice acceptor were tested. All 4 candidates yielded 100% editing of their target A in K562 cells (Table S1) and nearly 100% in primary human T cells from two healthy donors (Figure 5A and Table S1). To assess functional knockout, RNA was collected from stimulated primary T cells for RT-qPCR (Figure 5B). The exon 2 SA and exon 6 SD gRNAs reduced *NR4A3* transcript abundance by 0% and 57%, respectively. The two exon 1 SD guides differ only in the position of the target A within the protospacer sequence. When the target base was in position 9, a 98% reduction of *NR4A3* mRNA was observed. However, when the target base was in position 6, there was an 83% reduction in NR4A3 mRNA (Figure 5C).

DNMT3A is an enzyme that catalyzes 5-methylcytosine methylation and regulates T cell effector functions and differentiation early after activation.^58^ Figure 5D shows MultiEditRbatch results for exon 4 SD3 gRNA with 81% conversion of A–G at position 7, shown as T→C because the reverse primer was used for sequencing. Six candidate gRNAs nominated by SpliceR v.1.3.0 were screened in primary human T cells by digital western blot (Figure 5E). DNMT3A protein signal was normalized to signal from a β-actin control for comparison. Exon 4 SD gRNAs reduced expression more than exon 1 SD gRNAs, because exons 1 and 2 are untranslated regions, again demonstrating that targeting earlier exons is not always superior.

Granzyme A is a protease released from cytotoxic granules of immune effector cells that triggers cell death by interfering with the actin cytoskeleton of a target cell.^59^ Three candidate gRNAs were generated using SpliceR and the exon 1 SD resulted in the highest editing of 83% at position 5 in the gRNA protospacer (Figure 5G). Granzyme A is an example of a specialized protocol for detection of a secreted protein that is upregulated upon stimulation of immune cells. For secreted protein targets, we permeabilized edited primary T cells for intracellular staining treatment with protein transport inhibitors brefeldin A and monensin to allow accumulation of protein within the cell. To compare candidate gRNAs, each condition was performed with and without overnight stimulation with plate-bound OKT3 and CD28 stimulation to elicit production of cytolytic granules which contain GZMA. We observed a considerable reduction in GZMA production in edited cells (Figure 5H). As expected, edited cells also displayed an average 89.7% reduction in GZMA in two individual donors (Figure 5I). This underscores the importance of validating knockouts in appropriate conditions to model maximum expression of the target of interest.

## Discussion

Protein knockout via CRISPR-Cas9 has become an invaluable tool for both basic and translational research. Several companies provide user-friendly web-based platforms to design gRNAs, purchase reagents, and analyze resulting data in a streamlined manner. However, despite popularization of base editors, fewer platforms have emerged. This makes base editors less accessible for researchers outside of the genome engineering ecosystem. Many groups have highlighted pitfalls of CRISPR-Cas9 gene editing; the most extreme being unintended chromosomal losses and translocations, which are amplified when more than one gene is targeted simultaneously.^12^ Base editors have been demonstrated as a reliable tool for gene knockout without creation of DSBs, yet many researchers still gravitate toward CRISPR-Cas9 gene editing. It is our hope that the information in this manuscript makes the use of ABE for gene knockout more easily attainable for all scientists regardless of their field of study.

One of the most broadly used tools created to assist in gRNA design for base editors is BE-Hive (https://www.crisprbehive.design/guide).^37^ This tool uses machine learning algorithms to design gRNAs with the highest editing efficiencies and lowest abundance of unintended targets within the gRNA called bystander edits. Another application, SpliceR, nominates gRNAs and was built to complement BE-Hive by examining and ranking all possible splice-site mutations within a given transcript. It is important to note that SpliceR currently uses data from ABE7.10 and BE4max for its analysis, thereby reducing its accuracy in situations, where other versions of base editors are used. SpliceR could be improved by updating the calculation of ABE and CBE scores using data from currently used, more efficient enzymes, or by allowing selection of the specific base editor used for editing. Incorporation of machine-learning tools can and should also be used to further enhance the calculation of the cDNA disruption score by determining the probability of splice site mutations causing a frameshift in the spliced mRNA or by using protein level data such as alternative splicing products, known binding sites, phosphorylation sites, or critical enzymatic domains, which are available through online repositories such as UniProtKB.

Another application warranting machine learning tools is the prediction of gRNA secondary structure. Currently, secondary structure can be predicted using web tools, but aside from identifying the presence or absence of stem loops manually, there is not a way to definitively predict if the structure is likely to impede function. Figures 3 and Figure 1, Figure 2, Figure 3, Figure 4, Figure 5 further establish the differences in secondary structure predictions, their impact on editing, and the difficulty of manually interpreting predicted secondary structures. Furthering the predictive capabilities of gRNA nomination tools would not only allow for more efficient knockout but could lead to rational design of gRNAs to modulate protein function with more precision. This, combined with the continuing evolution of base editors with new PAMs,^60^ tunable editing windows,^17^ and the development of deaminase domains capable of transversion mutations^61^ could greatly increase the proportion of the genome that is amenable to base editing.

This manuscript also presents an updated application for analysis of sequencing data for base editing detection called MultiEditRbatch. Building upon the initial EditR application and subsequent MultiEditR, we sought to incorporate a batch mode for bulk analysis of data. A key improvement of MultiEditR was the incorporation of control files to compare differences in base frequency with statistical backing. This allowed for analysis of Sanger sequencing data with accuracy, precision, and reliability comparable to RNA sequencing and deep amplicon Next-Generation Sequencing. The subsequent version, called MultiEditRbatch, allows for bulk input and analysis. The MultiEditRbatch algorithm is poised to accommodate the evolving landscape of base editors. It is agnostic to the type of editor used and can detect multiple mutations within one protospacer motif. Input of the wild-type and edited bases allow for analysis of Sanger sequencing traces from experiments using ABE, CBE, transversion editors, and even installation of SNPs with prime editors. An important limitation of the MultiEditR algorithm to note is that it is not equipped to identify indels, so any enzyme suspected of inducing indels should also be analyzed using other indel-centric programs, such as Synthego ICE or TIDE (Tracking of Indels by Decomposition) analysis.

To demonstrate the workflow for implementing ABE for gene knockout, we validated gRNAs for ABE-mediated knockout of 14 immune relevant genes. Beyond serving as examples, these gRNAs may be useful for basic immunology research in applications, which are not amenable to DSBs. All but one of the selected gRNAs employs the ABE8e enzyme with the canonical spCas9 NGG PAM requirement. Some of these targets, such as IL-10Ra, also tested NG restricted PAMs which proved less efficient than the NGG gRNA candidates (Table S1 and Figure S2). The importance of comprehensive validation and rational gRNA design were demonstrated by the failed validation of perforin knockout gRNAs (Table S1 and Figure 3). The 14 validated guides use NGG base editors, which may have slightly stronger binding to the genomic DNA that could be the reason for the advantage over their NG counterparts as shown by the Granzyme A candidate gRNAs (Table S1).^18^^,^^31^ Therefore, when possible, it is best to use NGG PAM gRNAs for the highest editing efficiency and NG variants when NGG PAMs are not available.

One of the main advantages of ABE-based gene knockout is how amenable the system is to multiplex knockouts.^11^^,^^12^^,^^27^ Although ABE has been reported to create DSBs, it is at a significantly lower rate than Cas9 (up to ˜3% indel rate in hematopoietic stem cells reported by Fiumara et al.).^11^ Therefore, there are fewer if any opportunities for chromosomal translocations or losses that could lead to confounding results. Previously, the only way to avoid translocations was to knock out genes sequentially, which requires more time and reagents and may have a negative impact on cell health. It is important to keep in mind that only one enzyme variant should be used in each transfection experiment. The use of more than one base editor variant leads to a reduction of on-target efficiency due to gRNA exchange and potential for unintended edits.^30^^,^^62^ Recently, multiplexed orthogonal base editor (MOBE) systems have been developed to tether gRNAs to their appropriate enzymes using aptamers to reduce the effect of gRNA exchange.^63^ Further development and application of these MOBEs will enable use of multiple variants without reducing overall efficiency.

In conclusion, ABEs are an underutilized tool for gene knockout, owing partly to the lack of user-friendly platforms. Here, we clearly outline the process of target gene knockout with ABE from designing gRNAs using the SpliceR web-application to analysis. Representative results demonstrate multiple methods for validation of protein or transcript level knockout. Lastly, we debut MultiEditRbatch, a web-based application and R package for analysis of base-edited samples using Sanger sequencing. MultiEditRbatch offers robust analysis and a user-friendly web-based application.

## Materials and Methods

### Gene editing reagents

Guide RNAs were designed using SpliceR (https://moriaritylab.shinyapps.io/splicer/) or manually using SnapGene v.8.0.1. Alt-R CRISPR-Cas9 sgRNAs (Integrated DNA Technologies) were resuspended in nuclease-free H_2_O at 1 μg/μL for electroporation. Custom ABE8e and ABE8e-NG mRNAs were purchased from TriLink Biotechnologies (San Diego, CA) as modified mRNA transcripts containing pseudouridine and 5-methylcytidine and capped with CleanCap AG. Plasmids encoding ABE8e and ABE8e-NG (pmRNA_dT7_ABE8eNGG, and pmRNA_dT7_ABE8eNG respectively) were provided to TriLink Biotechnologies for *in vitro* transcription. Primers were used to mutate the dead T7 promoter and incorporate a 120A polyadenylated tail. Custom mRNA was treated with DNase I and phosphatase, purified using a silica membrane, and resuspended in 1 mM sodium citrate, pH 6.4, to a final concentration of 3 mg/mL by TriLink Biotechnologies.

### Cell culture

K562 cells were cultured in RPMI 1640 medium (Fisher Scientific, Cat #SH30027.01) supplemented with 10% heat-inactivated fetal bovine serum (FBS, Bio-Techne, Cat #S11550H) and 1% penicillin-streptomycin (Fisher Scientific, Cat #SV30031.01) and maintained at a density of 0.2 × 10^6^ to 1 × 10^6^ cells/mL. Primary human T cells and NK cells were isolated and cultured as previously described.^12^^,^^27^ Primary human cells were obtained after informed consent with approval from the University of Minnesota Institutional Review Board (IRB 1602E84302)

### Electroporation

Cells were engineered at ≥80% viability, as determined by Countess 3 FL automated cell counter (Fisher Scientific, Cat #AMQAF2000). Each electroporation reaction included 1 μg of sgRNA and 1.5 μg ABE8e or ABE8e-NG mRNA. HeLa cells were electroporated using the Neon Transfection System (Thermo Fisher Scientific) kit with 2 pulses of 1,005 V 35 ms with 0.5 × 10^5^ cells per 10 μL tip in R buffer. Primary NK cells were electroporated using the Neon Transfection System with 2 pulses of 1,825 V for 10 ms, using 0.3 × 10^6^ cells per 10 μL tip in T buffer, as previously described.^27^ K562 cells (0.2 × 10^6^ per reaction) were electroporated in 20 μL reactions using the Amaxa 4D nucleofector platform (Lonza) and the SE kit, with pulse code FF-120. as previously described, 1 × 10^6^ primary T cells per condition were electroporated using the P3 kit and pulse code EO-115 and cultured.^12^ Electroporated cells were cultured at least 3 days prior to assessing genomic editing, and 5 days prior to assessing functional knockout, to allow for mRNA and protein turnover.

### Assessment of genomic editing

Cell pellets containing 0.2–1 × 10^6^ cells were collected, and genomic DNA was isolated using the GeneJet genomic DNA extraction kit (Thermo Fisher Scientific, Cat #K0721). Primers were designed using Primer3 (v.4.1.0) and ordered as single-stranded DNA oligos from Integrated DNA Technologies.^64^ Amplicons spanning the editing site were PCR amplified using 100–200 ng of genomic DNA as template and AccuPrime Taq Polymerase (Invitrogen, Cat # 12346086). A portion of each PCR was analyzed by gel electrophoresis to confirm the presence of a single band of the expected size. PCR amplicons were purified using the QIAquick PCR purification kit (QIAGEN, Cat #28106) and submitted for Sanger sequencing to Eurofins Genomics (Louisville, KY, US). Sequencing results were returned as.ab1 files and analyzed using the previously published EditR (v.1.0.10) or our newly developed tool, MultiEditRbatch (v.1.0).

### Multiedit R R package

The MultiEditR algorithm, originally published as a standalone Shiny app (Kluesner et al., 2021), was implemented into an open-source, installable R package (GitHub:MoriarityLab/multiEditR.pckg). The package can be installed using the devtools package with the following command: devtools:install_github(“MoriarityLab/multiEditR.pckg”). As in the original app, this package depends on several R libraries for manipulating Sanger sequence data (sangerSeqR, Biostrings), statistical modeling (gamlss, gamlss.dist), and data wrangling and plotting (tidyverse). Briefly, the algorithm extracts nucleotide sequence from raw Sanger sequence data, aligns control and sample sequences, identifies the target motif and potential editing sites, and calculates the probability of editing based on the base call probabilities at editable positions. Users can specify key parameters, including the Phred score cutoff for base calling and the *p* value threshold for detecting base edits.

To perform a single comparison, users can call the primary function detect_edits(). This function takes as input: paths to the sample and control sequence files, the target motif (as a string), the base which may be mutated in the wt, the expected mutation of that base, and the use-defined Phred and *p* value cutoffs. detect_edits() returns a list containing a table of editing probabilities ($sample_data) and GAM statistical coefficients ($statistical_parameters). The user can visualize the chromatograms of the sample and control using plot_sample_chromatogram() and plot_control_chromatogram(), examine the normalized base call signals and noise using plot_raw_sample() and plot_trimmed_sample(), and, most usefully, view the base editing predictions in the sample motif with plot_editing_barplot().

To facilitate high throughput experiments, users can perform batch analysis using detect_edits_batch(), which takes a single argument: a data.frame() containing the same input fields as detect_edits(), but with different samples in different rows. An example parameters data frame can be obtained using load_example_params(), and a skeleton Excel file can be generated using save_batch_skeleton(“path/to/saved/file.xlsx”). The completed table can be imported into R using readxl:read_excel() and passed to detect_edits_batch(). After batch processing, result tables can be extracted using get_batch_results_table() and get_batch_stats_table(). Furthermore, an HTML report with figures and results tables for each prediction can be generated using create_multiEditR_report(), which requires the batch results from detect_edits_batch, the parameters, and a path where the report should be saved.

### MultiEditR batch mode shiny app

To complement the R package, we developed a Shiny app that enables users to run batch mode through a user-friendly web interface (https://moriaritylab.shinyapps.io/MultiEditRBatch/). As with the package, users can download skeleton spreadsheets to create parameter tables, which are then uploaded along with Sanger sequence files. The app runs detect_edits_batch() and returns downloadable results tables and an HTML report. To support users, the app includes step-by-step instructions with screenshots. The app is version-controlled and open-sourced in the Github repository (https://github.com/MoriarityLab/multiEditR.pckg).

### Protein-level validation of knockouts

Rested primary human T cells were stimulated for flow cytometry with either overnight with plate-bound CD3 (clone OKT3) and soluble CD28 (clone CD28.2) or for 4 h with PMA and ionomycin. Rested primary human NK cells were stimulated with IL-12 (10 ng/mL) and IL-18 (100 ng/mL). GolgiStop and GolgiPlug (BD Biosciences, Cat #51-2092KZ, 51-2301KZ) were added to enhance intracellular protein accumulation. Cells were stained using the BD Cytofix/Cytoperm kit (BD Biosciences), human TruStain FcX (BioLegend, Cat #422302), and antibodies listed in Table S3. Flow cytometry was performed on the CytoFlex S (Beckman Coulter) and analyzed with FlowJo software v.10.10.0. Digital western blotting was performed using antibodies listed in Table S4. Signal intensities were normalized using the ProteinSimple Jess system and analyzed with Compass for Simple Western analysis software v.7.0.0 (Bio-Techne). TaqMan gene expression assays were performed on a Bio-Rad CFX96 instrument and analyzed with CFX Maestro software v.5.3.022.1030, using probes listed in Table S5. Graphs were generated using GraphPad Prism (BD) v.10.4.1. BioRender was used to create Figure 1.

## Data and code availability

The MultiEditRbatch web app is available at https://z.umn.edu/multieditrbatch. Source code for running the application locally and recreating figures and analyses is available at https://github.com/MoriarityLab/multiEditR.pckg. All reasonable requests can be made to the corresponding author at mori0164@umn.edu.

## Acknowledgments

E.J.E. acknowledges funding from NIH grant F31AI181472. B.R.W. acknowledges funding from 10.13039/100000002NIH grants (R21CA237789, R21AI163731, P01CA254849, P50CA136393, R01AI146009, and U54CA268069); 10.13039/100000885Children’s Cancer Research fund,; 10.13039/100002039Cure Childhood Cancer; and the 10.13039/100013187Randy Shaver Cancer Research and Community Fund. B.S.M. acknowledges funding from the Office of Discovery and Translation, 10.13039/100000002NIH grants (R01AI146009, R01AI161017, P01CA254849, P50CA136393, U24OD026641, U54CA232561, P30CA077598, and U54CA268069, 10.13039/100000005DOD grants HT9425-24-1-1005, HT9425-24-1-1002, HT9425-24-1-0231); and 10.13039/100000885Children's Cancer Research fund, the 10.13039/100002086Fanconi Anemia Research fund, and the 10.13039/100013187Randy Shaver Cancer and Community Fund. M.K. acknowledges funding from 10.13039/100000002NIH grant F30CA305905-01. J.G.S. acknowledges support from MIB Agents and DOD grant HT9425-25-1-0744.

## Author contributions

E.J.E., M.K., M.J.J., B.R.W., and B.S.M. conceptualized the study; E.J.E., B.J.W., B.K., A.J.W., J.T.B., and M.W. performed experiments and analyzed the data; J.S.C. and M.K. developed the software which was validated by E.J.E. and A.J.W.; E.J.E. wrote the original draft with input from J.S.C., M.J.J., B.K., J.G.S., B.R.W., and B.S.M. participated in revisions and editing; B.R.W. and B.S.M. provided resources, obtained funding, and supervised.

## Declaration of interests

The authors declare no competing interests.

## Supplemental information

Document S1. Figures S1–S3 and Tables S2–S5

Table S1. All tested gRNAs See attached Excel. Includes Ensembl transcript ID used for design of gRNA with SpliceR, gRNA name, gRNA sequence, ABE variant used, SpliceR cDNA score, SpliceR ABE score, target base within gRNA, exon target, target strand, protein/mRNA loss %, method of protein/mRNA loss verification, genomic editing efficiency in K562 cell line and genomic editing efficiency in primary immune cells.

Document S2. Article plus supplemental information
