# Supplementary material for: Adenine base editor for knockout of proteins: A practical guide from design to analysis with updated MultiEditRbatch
Source: Mol Ther Nucleic Acids. 2026 Mar 18;37(2):102908. doi: 10.1016/j.omtn.2026.102908 (PMC13101598; doi:10.1016/j.omtn.2026.102908)
Supplement: Document S1. Figures S1–S3 and Tables S2–S5 [file mmc1.pdf]

OMTN, Volume *37*

Supplemental information

Adenine base editor for knockout of proteins: A practical guide from design to analysis with updated MultiEditRbatch

Ella J. Eaton, Bryce J. Wick, Jeremy S. Chacón, Amy J. Wang, Mitchell Kluesner, Jackson T. Barnes, Bibekananda Kar, Minjing Wang, Matthew J. Johnson, Joseph G. Skeate, Beau R. Webber, and Branden S. Moriarity

Table S1: All tested gRNAs. See attached Excel. Includes Ensembl Transcript ID used for design of gRNA with SpliceR, gRNA name, gRNA sequence, ABE variant used, SpliceR cDNA score, SpliceR ABE score, target base within gRNA, Exon target, Target strand, protein/mRNA loss %, method of protein/mRNA loss verification, genomic editing efficiency in K562 cell line and genomic editing efficiency in primary immune cells.

Table S2: qPCR assessment of transcript abundance. Cq values for qPCR runs.Target gene Cq is the average of 4 technical replicates. Percent loss of transcript is equal to 1 – (2 ^-ΔΔCq) where ΔΔCq = (Average Cq of treated target gene – Average Cq of treated GAPDH) / (Average Cq of control target gene – Average Cq of control GAPDH).

Target gene	Guide name	Average Cq of target gene	Average Cq of GAPDH	mRNA % loss
NR4A1	Ex. 5 SA 1	27.85	27.51	0%
NR4A1	Ex. 5 SD 1	30.37	27.95	78%
NR4A1	Ex. 6 SA 2	28.09	28.15	0%
NR4A1	Ex. 6 SD 1	28.01	27.66	0%
NR4A2	Ex. 2 SA 1	31.70	37.92	0%
NR4A2	Ex. 2 SD 1	31.97	32.01	80%
NR4A2	Ex. 3 SA 1	31.79	34.92	17%
NR4A2	Ex. 6 SD 1	32.51	34.28	68%
NR4A2	Ex. 7 SA 1	32.49	33.26	84%
NR4A2	Ex. 7 SA 3	32.30	33.30	81%
NR4A2	Ex. 7 SD 2	31.87	36.40	0%
NR4A2	Ex. 8 SA 1	31.24	33.33	60%
NR4A3	Ex. 1 SD 1	31.06	27.94	84%
NR4A3	Ex. 1 SD 2	33.40	27.36	98%
NR4A3	Ex. 2 SA 1	30.59	30.11	0%
NR4A3	Ex. 6 SD 1	29.44	27.72	58%

Table S3: Flow cytometry antibodies

Company	Catalog #	Target	Clone	Fluorophore
Biolegend	507207	Granzyme A	CB9	PacBlue
Biolegend	372208	Granzyme B	QA16A02	PE
Invitrogen	12977441	Granzyme M	4B2G4	PE
Biolegend	348010	Granulysin	DH2	APC
Biolegend	308104	Perforin	dG9	FITC
Biolegend	308803	IL10Ra	3F9	PE
Biolegend	328626	CD107a	H4A3	BV421
Invitrogen	65086518	eBioScience Fixable Viability Dye		eFluor780

Table S4: Western Blot antibodies

Company	Catalog #	Target	Clone
Cell Signaling Technology	46510S	HPK1	E1C3L Rabbit mAb
Cell Signaling Technology	55313S	SOCS1	E3Q4M Rabbit mAb
Sigma Aldrich	HPA035375	RASA2	polyclonal
Cell Signaling Technology	9498S	CBL-B	D3C12 Rabbit mAb
Cell Signaling Technology	14693S	PTPN22	D6D1H Rabbit mAb
Cell Signaling Technology	3598S	DNMT3A	D23G1 Rabbit mAb
Cell Signaling Technology	8457S	B Actin	D6A8 Rabbit mAb

Table S5: qPCR probes

Company	Catalog #	Target
Thermo Fisher Scientific	Hs00374226_m1	NR4A1
Thermo Fisher Scientific	Hs01117527_g1	NR4A2
Thermo Fisher Scientific	Hs00545009_g1	NR4A3

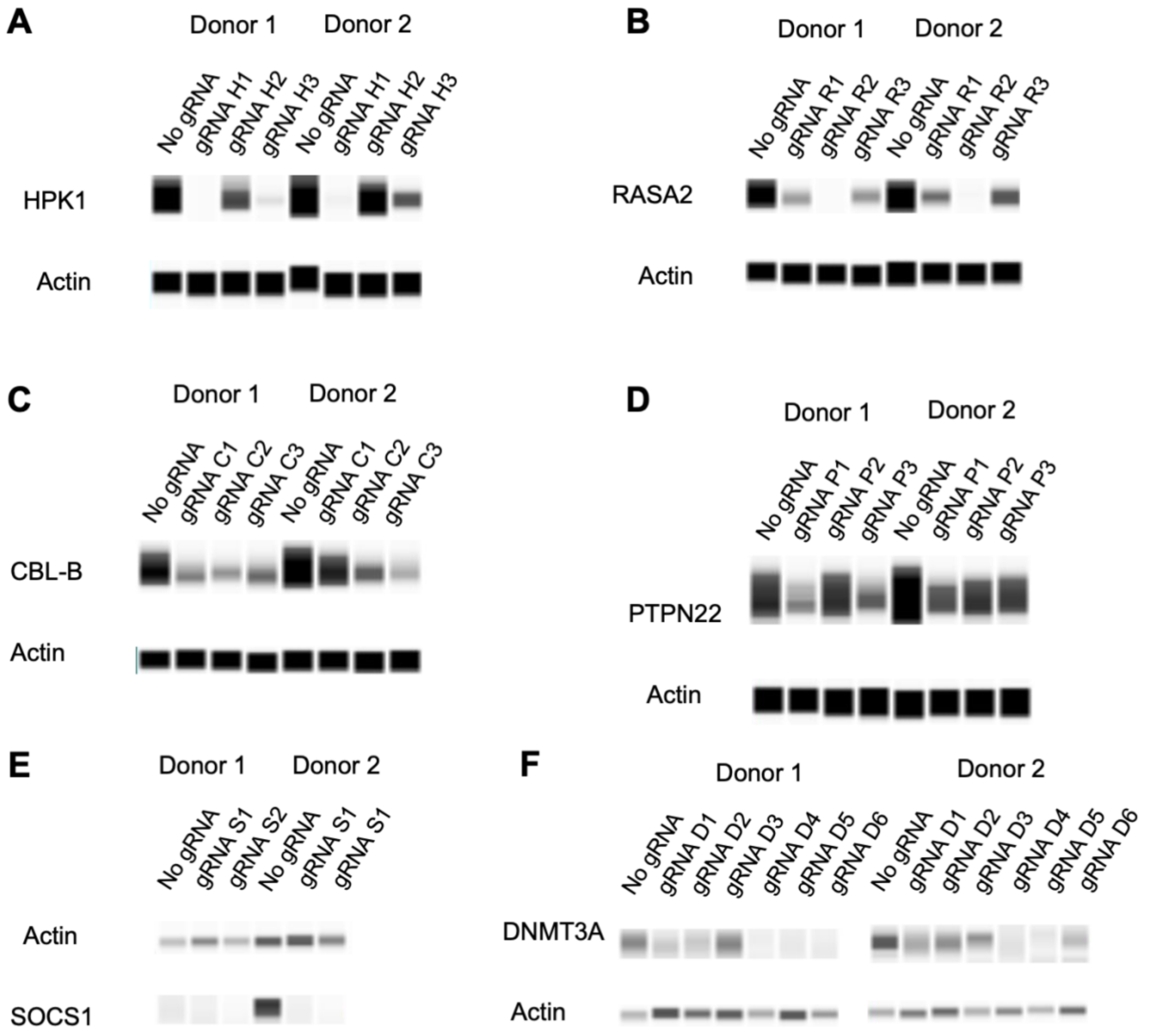

Figure S1: Western blot assessment of protein knockout. (A) HPK1 and Actin, (B) RASA2 and Actin, (C) CBL-B and Actin, (D) PTPN22 and Actin, (E) SOCS1 and Actin, (F) DNMT3A and Actin.

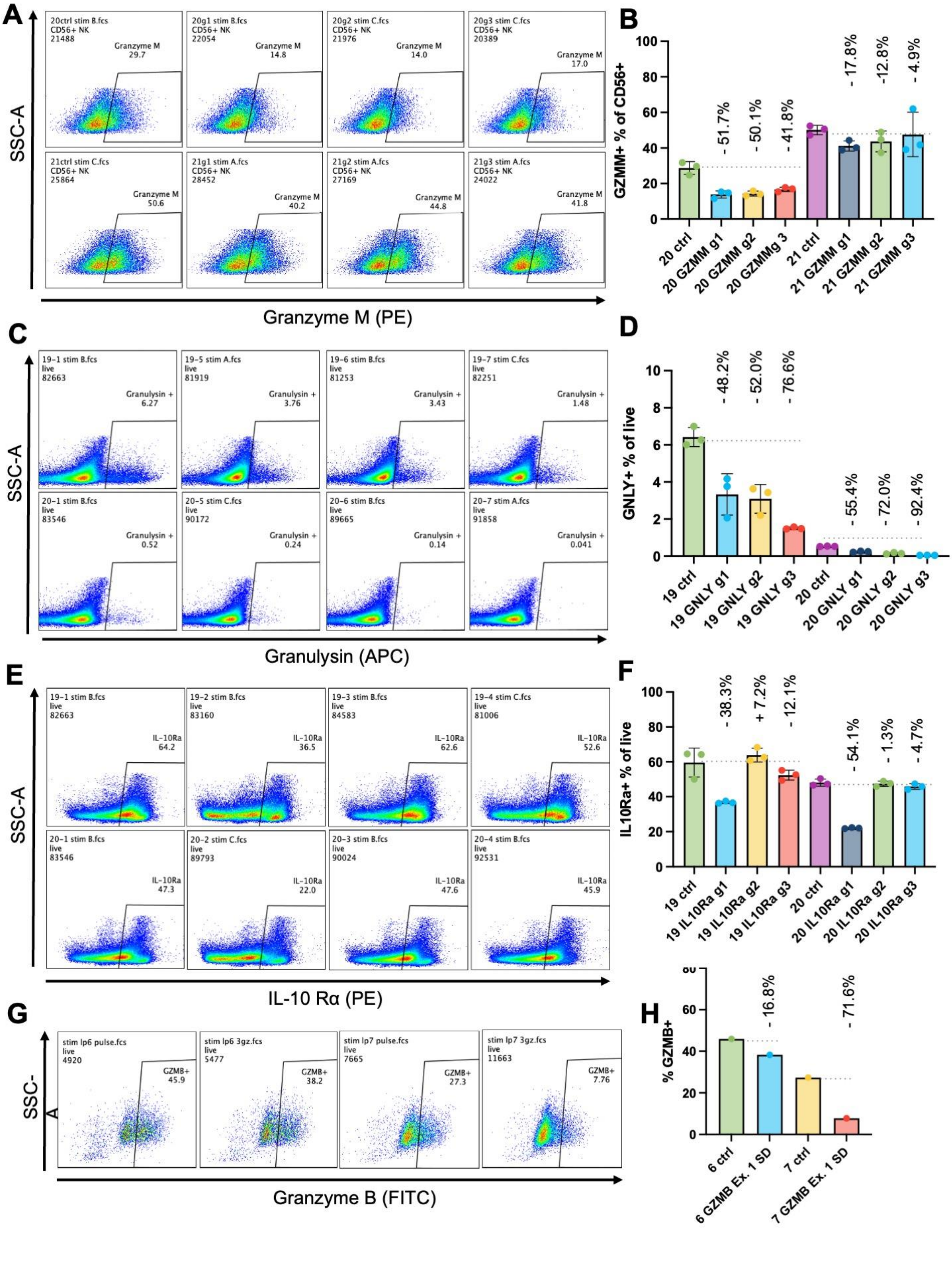
A
20ctrl stim B.fcs CD56+ NK 21488 Granzyme M 29.7
20g1 stim B.fcs CD56+ NK 22054 Granzyme M 14.8
20g2 stim C.fcs CD56+ NK 21976 Granzyme M 14.0
20g3 stim C.fcs CD56+ NK 20389 Granzyme M 17.0
21ctrl stim C.fcs CD56+ NK 25864 Granzyme M 50.6
21g1 stim A.fcs CD56+ NK 28452 Granzyme M 40.2
21g2 stim A.fcs CD56+ NK 27169 Granzyme M 44.8
21g3 stim A.fcs CD56+ NK 24022 Granzyme M 41.8
SSC-A
Granzyme M (PE)
B
GZMM+ % of CD56+
- 51.7%
- 50.1%
- 41.8%
- 17.8%
-12.8%
- 4.9%
20 ctrl
20 GZMM g1
20 GZMM g2
20 GZMMg 3
21 ctrl
21 GZMM g1
21 GZMM g2
21 GZMM g3
C
19-1 stim B.fcs live 82663 Granulysin + 6.27
19-5 stim A.fcs live 81919 Granulysin + 3.76
19-6 stim B.fcs live 81253 Granulysin + 3.43
19-7 stim C.fcs live 82251 Granulysin + 1.48
20-1 stim B.fcs live 83546 Granulysin + 0.52
20-5 stim C.fcs live 90172 Granulysin + 0.24
20-6 stim B.fcs live 89665 Granulysin + 0.14
20-7 stim A.fcs live 91858 Granulysin + 0.041
SSC-A
Granulysin (APC)
D
GNLY+ % of live
- 48.2%
- 52.0%
- 76.6%
- 55.4%
- 72.0%
- 92.4%
19 ctrl
19 GNLY g1
19 GNLY g2
19 GNLY g3
20 ctrl
20 GNLY g1
20 GNLY g2
20 GNLY g3
E
19-1 stim B.fcs live 82663 IL-10Ra 64.2
19-2 stim B.fcs live 83160 IL-10Ra 36.5
19-3 stim B.fcs live 84583 IL-10Ra 62.6
19-4 stim C.fcs live 81006 IL-10Ra 52.6
20-1 stim B.fcs live 83546 IL-10Ra 47.3
20-2 stim C.fcs live 89793 IL-10Ra 22.0
20-3 stim B.fcs live 90024 IL-10Ra 47.6
20-4 stim B.fcs live 92531 IL-10Ra 45.9
SSC-A
IL-10 Rα (PE)
F
IL10Ra+ % of live
- 38.3%
+ 7.2%
- 12.1%
- 54.1%
- 1.3%
- 4.7%
19 ctrl
19 IL10Ra g1
19 IL10Ra g2
19 IL10Ra g3
20 ctrl
20 IL10Ra g1
20 IL10Ra g2
20 IL10Ra g3
G
stim lp6 pulse.fcs live 4920 GZMB+ 45.9
stim lp6 3gz.fcs live 5477 GZMB+ 38.2
stim lp7 pulse.fcs live 7665 GZMB+ 27.3
stim lp7 3gz.fcs live 11663 GZMB+ 7.76
SSC-A
Granzyme B (FITC)
H
% GZMB+
- 16.8%
- 71.6%
6 ctrl
6 GZMB Ex. 1 SD
7 ctrl
7 GZMB Ex. 1 SD

Figure S2: Flow cytometric assessment of protein knockout. (A) Representative flow plots of 2 independent donors of primary NK cells. Gating scheme: Lymphocytes, single cells, live, CD56+. Controls for each donor which received no gRNA on left, followed by gRNA 1-3. (B) Percent of Granzyme M + of CD56+ cells, dots represent technical replicates of intracellular staining. Dotted line shows average expression for control for each donor. Difference from the control, or percent loss of expression is noted above each bar and is equal to 1 - (average of each group / average of control) * 100. (C) Representative flow plots of 2 independent donors of primary T cells. GNLY gating scheme: Lymphocytes, single cells, live, CD3+. Controls for each donor which received no gRNA on left, followed by gRNA 1-3. (D) Percent of Granulysin+ of CD3+ cells, dots represent technical replicates of intracellular staining. (E) Representative flow plots of 2 independent donors of primary T cells. IL-10Rα gating scheme: Lymphocytes, single cells, live, CD3+. Controls for each donor which received no gRNA on left, followed by gRNA 1-3. (F) Percent of IL-10Rα + of CD3+ cells, dots represent technical replicates of intracellular staining. (G) Flow plots of 2 independent donors of primary T cells. Gating scheme: Lymphocytes, single cells, live, CD3+. Left to right: donor #6 control, donor #6 GZMB Ex. 1 SD, donor #7 control, donor #7 GZMB Ex. 1 SD. (H) Percent of Granzyme B + of CD3+ cells, n=1.

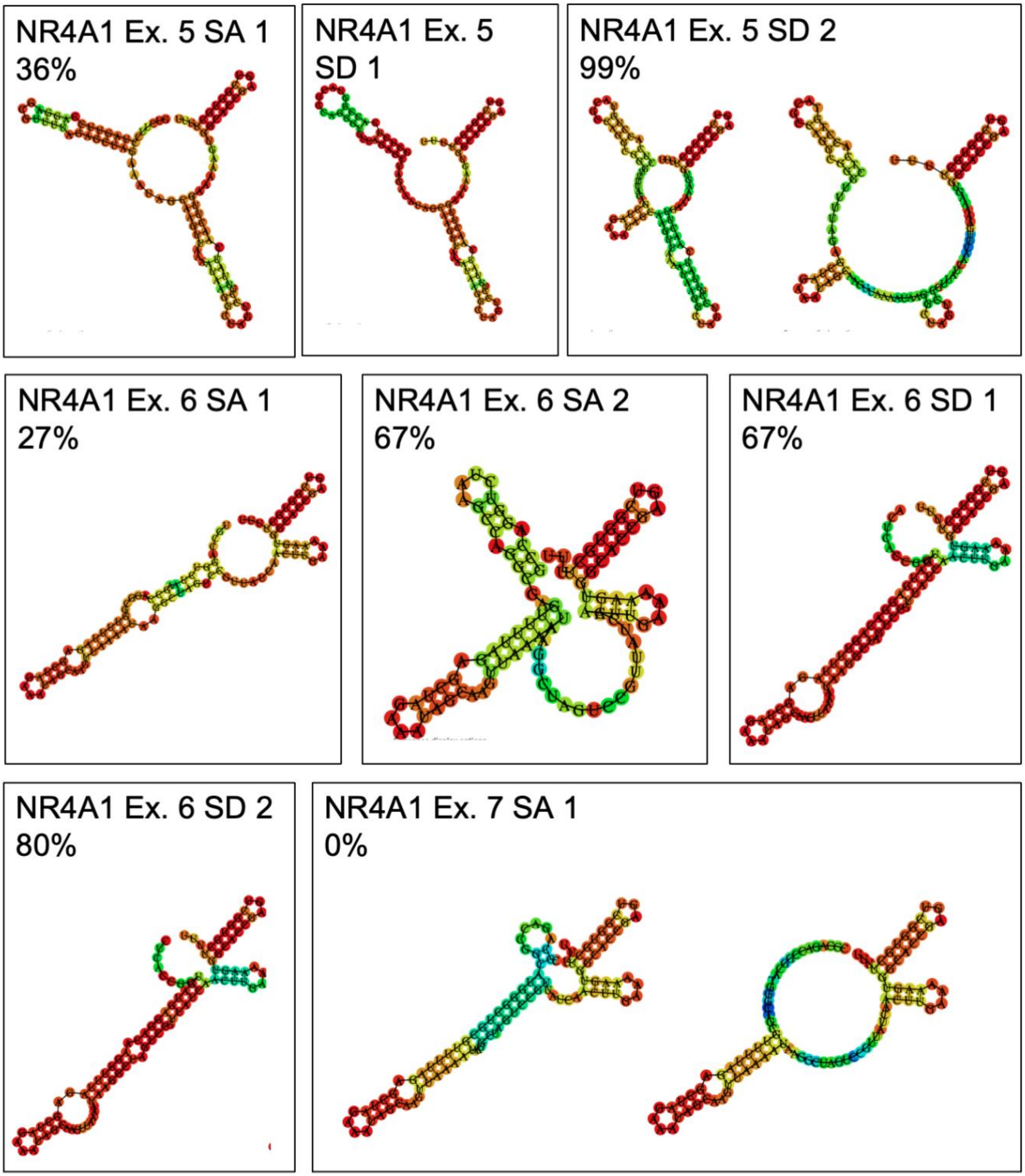

Figure S3: RNAfold prediction of gRNA secondary structure. The RNAfold web server was used to predict secondary structure of gRNAs for NR4A1. Each gRNA name is accompanied by the editing efficiency in the K562 cell line. RNAfold input was the 20-nucleotide gRNA protospacer followed directly by the standard 80-nucleotide scaffold (5'GUUUUAGAGCUAGAAAUAGCAAGUUAAAAUAAGGCUAGUCCGUUAUCAACUUGAAAAAGUGGCACCGAGUCGGUGCUUUU3').
